# Strengthening the capabilities of families and communities to improve child health in low and middle income countries

**DOI:** 10.1136/bmj.k2649

**Published:** 2018-07-30

**Authors:** Audrey Prost, David Sanders, Anthony Costello, Joanna Vogel, Abdullah H Baqui, Nirmala Nair, Magali Romedenne, Ketan Chitnis, Geoffrey Bisoborwa, Tanya Doherty

**Affiliations:** 1 London School of Hygiene and Tropical Medicine, UK; 2 School of Public Health, University of the Western Cape, South Africa; 3 Department of Paediatrics and Child Health, University of Cape Town, South Africa; 4 Department of Maternal, Child and Adolescent Health, World Health Organization, Geneva, Switzerland; 5 Johns Hopkins Bloomberg School of Public Health, Baltimore, Maryland, USA; 6 Ekjut, Chakradharpur, India; 7 Unicef West and Central Africa Regional Office, Dakar, Senegal; 8 Unicef, New York, USA; 9 World Health Organization, Regional Office for Africa, Brazzaville, Republic of Congo; 10 Health Systems Research Unit, South African Medical Research Council, Cape Town, South Africa

## Abstract

**Audrey Prost and colleagues** discuss how best to enable families and communities to improve child health

The concept of community involvement in delivery of healthcare gained prominence after the 1978 Alma Ata conference on primary care.^1^ Many countries established large scale community health worker programmes with paid or voluntary workers to provide preventive, promotive, and curative care focusing on women and children. 

During the millennium development goal period, the HIV/AIDS epidemic in sub-Saharan Africa, combined with generalised shortages of skilled health personnel in low and middle income countries,^2^ increased the need for health workers. This resulted in persistently low coverage of high impact interventions for children and growing equity gaps within and between countries.^3^ The involvement of families and communities has emerged as important for increasing access to health services, particularly for those in rural and hard to reach areas.^4^ Health providers can support families to provide adequate home care for children’s healthy growth and development. Families also need to be able to respond appropriately when children are sick, seek appropriate timely assistance, and follow recommended treatments.

The global Integrated Management of Childhood Illness (IMCI) strategy was launched by WHO and Unicef in 1997. It included a component focusing on communities, known as community IMCI or C-IMCI. This component advocated 16 key family practices to improve maternal and child health (box 1). These were expanded to provide detailed recommendations in the *Facts for life* booklet.^5^ However, implementation has been uneven.^6^ ^7^

Box 1 **Key family practices for improving child health and nutrition**

**For physical growth and mental development**

- Breastfeed infants exclusively for at least four months and, if possible, for up to six months. (Mothers found to be HIV positive require counselling about possible alternatives to breastfeeding)
- Starting at about 6 months of age, feed children freshly prepared energy and nutrient rich complementary foods, while continuing to breastfeed up to age 2 years or longer
- Ensure that children receive adequate amounts of micronutrients (vitamin A and iron, in particular), either in their diet or through supplementation
- Promote mental and social development by responding to a child’s needs for care through talking, playing, and providing a stimulating environment

**For disease prevention**

- Take children, as scheduled, to complete a full course of immunisations (BCG; diphtheria, tetanus, and pertussis; oral polio vaccine, and measles) before their first birthday
- Dispose of faeces, including children’s faeces, safely; and wash hands after defecation, before preparing meals, and before feeding children
- Protect children in malaria endemic areas by ensuring that they sleep under insecticide treated bed nets
- Adopt and sustain behaviour for prevention of HIV/AIDS and care of people affected, including orphans

**For appropriate home care**

- Continue to feed and offer more fluids, including breast milk, to children when they are sick
- Give sick children home treatment for infections
- Take actions to prevent and manage child injuries
- Prevent child abuse and neglect, and take action when it has occurred
- Ensure that men participate in providing child care and are involved in the reproductive health of the family

**For seeking care**

- Recognise when sick children need treatment outside the home and seek care from providers
- Follow the health worker’s advice about treatment, follow-up, and referral
- Ensure that every pregnant woman has adequate antenatal care. This includes having at least four antenatal visits with a healthcare provider and receiving the recommended doses of the tetanus toxoid vaccine. The mother also needs support from her family and community in seeking care at the time of delivery and during the postpartum and lactation period

A recent modelling study found that strengthening the delivery of key maternal, newborn, and child health interventions in the community, with 70% coverage, could have prevented an estimated 4.9 million deaths between 2016 and 2020, with the greatest benefits in the African region.^8^ Community involvement is therefore critical to achieving the sustainable development goal target 3.2—namely, reducing neonatal mortality to ≤12 per 1000 live births, and mortality of the under 5s to ≤25 per 1000 live births. Participation of communities in improving health also has broader societal benefits. It can enhance the accountability of health services towards the communities they serve, reduce expenditure for families by bringing care closer to them, and decrease poverty by providing paid employment for women as community health workers.^9^

We analysed available IMCI data, conducted a literature review, and interviewed 20 global informants (box 2) to assess the reasons for uneven progress in implementing C-IMCI, and determine what needs to be done next.

Box 2 **Methods and data sources**

Our methods are based on the 2016 strategic review coordinated by WHO and Unicef. The review aimed to summarise lessons learnt from 20 years’ implementation of Integrated Management of Childhood Illness (IMCI) strategy and to recommend options for future child health strategies.^10^ It incorporated qualitative and quantitative data sources, including interviews with global experts, country assessments, and literature reviews. A full description of methods has been provided elsewhere.^11^

For this article, we relied on the IMCI implementation survey of 95 countries,^10^ 20 global key informant interviews, 9 in-depth country assessments, and a literature review of community approaches to improving child health.

For the literature review we searched PubMed, CINAHL and Embase for articles with abstracts that included a key term (“community-integrated”, “community-based management of childhood illness”, “community engagement”, “community participation”, “behaviour change communication”, “home visit”, “groups”, “health committees”, “health days”, “child health days”, “family practices”, “care practices”) together with a term related to child health outcomes (“child survival”, “child development”, “child mortality”, “neonatal mortality”, “diarrhoea”, “pneumonia”, “malaria”). We searched for articles published between 2000 and 2016 and used the Cochrane filters for low and middle income countries. We included intervention studies using experimental and quasi-experimental methods, as well as systematic and non-systematic reviews. We selected community approaches according to their effects on child, infant or neonatal mortality, care seeking and/or homecare practices for children.

We reviewed qualitative and quantitative data specifically relating to community approaches and analysed them though further triangulation and discussion.

## What happened to C-IMCI?

A 2003 review of IMCI found that 103 countries were fulfilling its first two components—strengthening health workers’ skills and health systems for child health. However, only half had activities to improve family and community practices.^12^ Of 90 countries in a 2016 IMCI implementation survey, 77 (86%) reported C-IMCI activities. The most commonly used activities were home visits for counselling on some of the 16 key family practices (78%), visits in the neonatal period (70%), visits during pregnancy (66%) and activities with community groups (66%).^10^ These activities were often labelled “C-IMCI” because they took place in the community. However, country case studies showed that their nature and coverage were often determined by donor funded initiatives through non-governmental organisations (NGOs) and were often carried out in isolation from other community and facility initiatives. As a state public health official in Nigeria said: “there has to be a linkage between facilities and communities ... but there is no such linkage, rather there’s a vertical programme concerning community IMCI.”

We identified several factors that impeded the scale up and use of C-IMCI. Firstly, countries were unclear about the best approaches for promoting key family health practices for child survival, growth, and development. A 2001 implementation framework provided critical guiding principles for C-IMCI but was deliberately non-prescriptive in order to build on countries’ own experiences.^13^ This unusual lack of prescriptive “guidelines” from agencies may have inadvertently reduced the value of C-IMCI. One senior manager of a multilateral organisation said: “We were naive in thinking the community component could do the family practices. The laundry list of components and behaviours was not an effective mechanism of communication. While we’ve paid lip service [to community engagement] we haven't addressed it beyond distributing bed nets.” 

Secondly, the scale up of C-IMCI was hindered by a lack of investment in training, incentives, and supervision of community health workers. This was coupled with substantial reliance on funds from bilateral or multilateral agencies and NGOs for community level activities, which led to a lack of coordination. The 2016 IMCI implementation survey found that only 33% countries financed training and daily allowances for C-IMCI activities through government spending. In all other countries, these costs were largely met by bilateral and multilateral agencies. Furthermore, only 55% of countries provided community health workers with salaries or incentives. 

In some countries government community health workers were not incorporated in the wider health system to support C-IMCI and relied mainly on NGO provision. One key informant from Bangladesh explained that village health workers who are not regularly present in the community and linked to government health facilities may not be effective: “To promote those [family and community] practices you need somebody to be there in the village to have regular contact with the mother and the family. BRAC (an international development organisation based in Bangladesh) did it but that cadre is not in the government system. For scale up, we need [a] village-based volunteer or worker who is not there in the government system at present.” 

Finally, the three original components of IMCI were designed to be implemented together, but funding shortages and changing priorities often made this challenging. For example, improving the skills of health workers was often prioritised at the start of IMCI, with less investment in community activities. After the introduction of integrated community case management, there was renewed focus on community activities, but at the same time financial aid for linkages to facilities and the clinical component of IMCI was reduced. As a child health officer of an NGO in Bangladesh said: “Government is giving more emphasis on training of staff and supply of equipment and drugs to community clinics but not giving much attention to proper implementation of IMCI corners **(**separate areas for the management of sick children in subdistrict health facilities) in facilities.” All strategic reviews of countries highlighted the need to use the three IMCI components together to achieve results.

## What works?

More than two decades after the inception of IMCI, we have substantial evidence on effective community approaches to improve child health.

The 2.6 million neonatal deaths that occur annually constitute 46% of deaths among children under 5. The number of stillbirths is equally high.^14^ Many community approaches in the past 20 years therefore focused on reducing deaths and illness during the perinatal period. In 2015, Lassi et al reviewed 18 trials of community approaches, including home visits and working with women’s groups. They found clear benefits, with a 25% reduction in neonatal mortality and a 19% reduction in stillbirths.^15^

Home visits during pregnancy and the postnatal period were commonly reported by countries in the WHO strategic review. A meta-analysis of four effectiveness trials of home visits during pregnancy or the postnatal period conducted in Bangladesh, Pakistan, India, and Ghana under real world conditions, reported a 12% reduction in neonatal mortality.^15^ Two recent economic modelling studies have found that perinatal home visiting is likely to be cost effective in low and middle income countries, even under a range of neonatal mortality rate assumptions.^16^ ^17^

In view of increasing evidence, WHO and Unicef’s 2015 package “Caring for newborns and children in the community” recommends at least two routine visits in pregnancy, three in the first month of life, and three visits from 2 to 6 months postnatally.^18^ Evidence suggests that home visits can also be an effective opportunity to promote infection control, good feeding practices for infants and young children, immunisations, and early childhood stimulation, all of which are essential for growth and development.^19^ ^20^ In addition, many home visits now also involve detection, treatment, or referral for malaria, pneumonia, and diarrhoea, with synergies between these activities and promotion by community health workers of key family practices.^21^

Working with women’s groups is another effective approach to strengthening the ability of families and communities to improve child health. These approaches vary from message based health promotion to participatory, community-wide mobilisation. In the “care group” model, for example, 10-15 women meet monthly to learn health messages. They then relay these to 10 pregnant women or mothers of children under 5.^20^ A quasi-experimental study in five countries found that this method increased the coverage of several key child survival interventions.^21^ A more interactive approach involves groups that discuss beneficial behaviours and how to overcome barriers to practising them, usually with a facilitator. Such interventions have led to large reductions of neonatal mortality in trials when coupled with home visits, though benefits were fewer in “real world” programme settings than in smaller trials.^22^ ^23^

Groups can catalyse individual and community action for child health. One review included seven trials where women’s groups took part in a cycle of participatory learning and action meetings. It found that these led to a 20% reduction in neonatal mortality by promoting beneficial behaviours and building the ability of women and communities to act on selected social determinants of health.^24^ This review also found that women’s groups practising participatory learning and action were highly cost effective according to WHO recommended standards.^24^ In 2014, WHO recommended that learning and action cycles should be facilitated with women’s groups to improve maternal and newborn health in settings with low access to services.^25^

Participatory women’s groups meetings and home visits are cost effective strategies for improving neonatal survival in settings of mid-level (neonatal mortality rate 33-43/1000) and high mortality (≥44/1000). However, these approaches may be less effective at lower mortality levels (<32/1000). A recent meta-analysis of 17 trials, including home visits or women’s groups, reported a 25% reduction in neonatal mortality in high mortality settings, 11% reduction in places with mid-level mortality levels, and no evidence of effect in lower mortality areas. The authors concluded that the approach to community interventions to improve neonatal survival may need to be re-examined in lower mortality situations. However, they also noted other benefits beyond survival. These included enhanced health literacy, increased social support, better preparedness for birth, reduced delays in seeking care, and improved linkages between the community and health facilities for referrals.^26^

Other community approaches to improving child health during and beyond the perinatal period include training and discussion with husbands, partners, community leaders, and health committees. Discussion with men, leaders, and community health providers is widely used and has been credited with improving the effectiveness of integrated community case management in Niger, Mozambique, and Malawi.^27^ ^28^ A recent meta-analysis of 14 studies found that involving men in individual or group interventions increased the use of maternal health services, including skilled birth attendance and postnatal care. It led to a 66% reduction in the likelihood of postpartum depression (odds ratio 0.34, 95% CI 0.19 to 0.62; five studies).^29^

Evidence is increasing that health committees (for example, village health committees or health facility committees) improve demand for, and access to, services and good quality care.^30^ These committees usually comprise community representatives, community health workers, and facility based providers. They collect and monitor health data at a community level in order to enhance social accountability and enable better planning and decision making for healthcare provision.^30^ A systematic review of four studies found that health facility committees can effectively improve the quality and coverage of health services, as well as health outcomes.^30^ A recent Malawian trial of an approach to boost social accountability through community score cards found improvements in the proportion of women receiving antenatal and postnatal home visits, as well as improvements in overall satisfaction with the service.^31^

## What next?

Much remains to be done to improve children’s health in this era of sustainable development goals. Many effective interventions can and should be delivered through community involvement. Accumulating evidence shows the effectiveness of a range of approaches in improving child health. Lessons from this review suggest that scaling up such approaches requires coordinated planning; sustained funding linked to monitoring of activities; country led processes to identify locally appropriate content, coverage, and mix of community engagement approaches; and better coordination with facility based efforts.

To deal with the difficulty in scaling up community engagement approaches to promote key family practices under C-IMCI, WHO and Unicef released the publication, *Caring for Newborns and Children in the Community*. This describes a set of tools to help community health workers support practices for newborn health, children’s healthy growth and development, and the management of sick children in the community.^18^ This package currently focuses on home visits to caregivers and needs to be adapted for use with women’s and community groups, as these are common and important forums for community involvement. In scaling up strategies to involve the community, priority could be given to the most underserved areas and those with high mortality. This should be complemented with coordinated measures to improve the provision of quality, respectful care in facilities. As countries increase coverage of hospital births and neonatal mortality rates fall, community strategies will need to strengthen home care and care seeking for healthy growth and development within, but also beyond, the perinatal period.^32^

**Fig 1:**
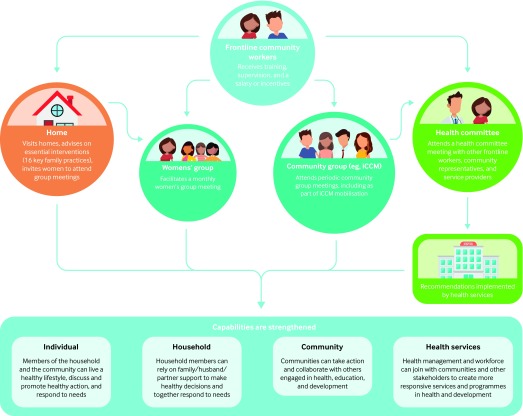
Suggested framework for community engagement activities to build the capabilities of individuals, families, and communities. iCCM=integrated community case management

Finally, there is a need to support country leadership and coordinated investment for community involvement strategies, linking with non-health sections that influence newborn and young child health, and for a clear set of monitoring indicators to foster accountability. Without country led coordination, NGOs and donors may continue to promote near vertical, isolated community engagement activities across the continuum of care for women, children, and adolescents. Figure 1 shows a possible framework for community engagement activities to build the capabilities of individuals, families, and communities within the context of WHO’s global strategy. Implementation research and learning networks between countries could facilitate community practice and facilitate scale up.^33^

**Key messages**

- Activities that help families and communities to improve child health are key to achieving the third sustainable development goal
- Evidence based approaches include counselling through home visits, participatory learning and action with women’s groups, training, and discussion with husbands, partners, community leaders, and health committees
- Country led, coordinated investment is required to develop strategies for involving the community linked with monitoring to facilitate accountability

## Notes

See www.bmj.com/child-health for other articles in the series.

Contributors and sources: AP wrote the original report on C-IMCI for the WHO strategic review of IMCI and the first draft of this article. TD and DS reviewed the structure of the article and wrote additional sections. All authors reviewed, critiqued and approved the final version of the article. The views expressed in this article do not necessarily represent the views, decisions, or policies of the institutions to which the authors are affiliated.

Provenance and peer review: Commissioned; externally peer reviewed.

This article is part of a series based on findings from a global review of Integrated Management of Childhood Illness funded by the Bill and Melinda Gates Foundation. Open access fees were funded by the Health Systems Research Unit, South African Medical Research Council. *The BMJ* peer reviewed, edited, and made the decision to publish the article with no involvement from the Medical Research Council.
